# An Easy Method of Bathing in Typhoid Fever

**Published:** 1894-09

**Authors:** W. B. Noyes


### AN EASY METHOD OF BATHING IN TYPHOID FEVER.

Every new medical text-book and periodical accumulates statistics testifying to the brilliant results following the use of cold baths in typhoid fever. The hospitals in which this method is chiefly carried on are almost, without exception, showing a higher percentage of recoveries than ever before under any other plan of treatment.

Why is it, then, that this method is not universally adopted and carried out in private practice? The answer is simple.

Easy as it is in a hospital with an abundance of skilled assistance, there is no method of treatment in use so difficult to carry out properly as tubbing in typhoid fever in private families.

As the family bath-room is generally out of the question, on account of its inconvenient location, and use by others of the household, it becomes necessary to buy a large portable bath-tub.

Even in cases where there are two trained nurses, it is necessary to call in the clumsy assistance of some members of the family to help in the lifting of the patient to and from the tub, and this is a strain on them, and not unattended with risk to the one who is sick.

There is, undoubtedly, a widespread opposition among the laity, both educated and uneducated, to the use of "cold baths" in fever. A young physician must have a very strong hold on the family, and enjoy their most complete confidence, to take the risk of the responsibility, if the disease takes a bad turn later from any accidental complications, and even older men will sometimes hesitate.

A physician of high standing and extensive practice in the city recently said to me: "I do not find that tubbing is practicable in private families, much as I would like to make use of it. I have to content myself with sponging and packs, and have had some success with constant use of an ice-coil over the abdomen as a substitute. If the ice-coil acts so well in lesions of the peritoneal coat of the intestines, why should it not have a similar result in lesions of the mucous coat?"

But all these three methods, sponging, wet pack or ice-coil, while they add to the comfort of the patient, do not cause very decided or long-continued lowering of the temperature.

Again, one who has had bad results from necessary changing of the position of a patient in an advanced stage of typhoid, to examine the back of the chest or something equally simple, causing disagreeable heart symptoms, will rather dread the process of lifting a patient out of his bed for any reason whatever, especially if the patient is large and the bed a double one.

The following method, which I once saw used in a hospital in Buffalo, commends itself as a simple solution of the whole problem. I have never seen it published in any medical journal or work, and am sure that it has not often been tried.

It is a very easy thing to slip a rubber blanket under the patient, and raise the two sides and the ends at the foot and head of the bed, nine or ten inches, by a row of pillows, bolsters, sand-bags or simple boards.

The rubber blanket ought to be of double thickness, as large as can be purchased, and special care must be given to the arranging of the corners. When this is done, you have the patient at the bottom of an impromptu bath-tub, into which you can pour water at any desired temperature, and in sufficient quantity to partially or entirely cover his body.

Only two inches of water would be enough to give a cool sponge-bath ten times as efficacious as the gingerly sponging possible under ordinary circumstances, and if the sides and corners are firmly fixed, you can easily make this tub hold all the water you desire. The water may be run from the nearest faucet by a rubber tube. I have found it a simpler and equally successful method to carry it in pails and pour it over the patient, starting with tepid and gradually cooling it down to the desired temperature.

The neatest method I have found by experiment is to use a large "watering-pot" with a sprinkler, such as is used for watering plants. This is a method which will not commend itself to those who dislike humble and commonplace methods to accomplish something that more complicated and more impressive methods might do.

I believe this kind of a bath will always be grateful to the patient, and if it is found necessary to use water at a decidedly low temperature, would give rise to less shock than a sudden plunge into a tub filled with very cold water. The effect on the temperature is the same as it would be under the other method with a stationary tub.

The water can be removed, without spilling a drop on the bed, by siphoning with a rubber tube, or dipping with a small pitcher or cup or sponging. Then the blanket can be dried and left in place, covered by a clean sheet, or, better yet, removed and dried in the sun.

All this needs a little care, for it would be a very serious and troublesome accident to soak the patient's bed-clothing and mattress, but the difficulty and risk to the patient cannot be compared with that attending the use of the ordinary tub. And, best of all, in those cases where the family or friends must do the work, and the employment of trained nurses is impossible on account of expense, this method can always be successfully used when tubbing would be out of the question.

I have not found one single objection to the employment of the method in private cases, and cannot see why it could not be used in hospitals.—*Dr. W. B. Noyes in Medical Record.*

